# Monoclonal Antibodies for Non-Hodgkin's Lymphoma: State of the Art and Perspectives

**DOI:** 10.1155/2010/428253

**Published:** 2011-03-06

**Authors:** Giulia Motta, Michele Cea, Eva Moran, Federico Carbone, Valeria Augusti, Franco Patrone, Alessio Nencioni

**Affiliations:** Department of Internal Medicine, University of Genoa, Room 221, V.le Benedetto XV 6, 16132 Genoa, Italy

## Abstract

Monoclonal antibodies have been the most successful therapeutics ever brought to cancer treatment by immune technologies. The use of monoclonal antibodies in B-cell Non-Hodgkin's lymphomas (NHL) represents the greatest example of these advances, as the introduction of the anti-CD20 antibody rituximab has had a dramatic impact on how we treat this group of diseases today. Despite this success, several questions about how to optimize the use of monoclonal antibodies in NHL remain open. The best administration schedules, as well as the optimal duration of rituximab treatment, have yet to be determined. A deeper knowledge of the mechanisms underlying resistance to rituximab is also necessary in order to improve the activity of this and of similar therapeutics. Finally, new antibodies and biological agents are entering the scene and their advantages over rituximab will have to be assessed. We will discuss these issues and present an overview of the most significant clinical studies with monoclonal antibodies for NHL treatment carried out to date.

## 1. Introduction

In 1975, Kohler and Milstein heralded a new era in antibody research with their discovery of hybridoma technology [1]. Mouse hybridomas were the first reliable source of monoclonal antibodies. Subsequently, the introduction of recombinant technologies, transgenic animals, and phage display technology has modernized selection, humanization and production of therapeutic antibodies. The use of mAbs in cancer treatment stems from the idea that these, because of their intrinsic specificity, could be used to selectively target cancer cells based on the expression of one or more antigens. In such approaches, antibodies could be used alone or be conjugated to toxins, radioactive moieties, or enzymes in order to achieve toxic concentrations of these agents in the cancerous tissues while sparing healthy organs.

Indeed, since their initial discovery, more than 20 mAbs have been approved by the US Food and Drug Administration (FDA) for the treatment of several conditions, including several types of cancers. This success has opened new therapeutic perspectives and prompted research efforts aimed to improve their activity, select for those patients who will most benefit from them, and, potentially, to expand their therapeutic indications. The anti-CD20 mAb rituximab is one of the best examples of this new class of therapeutics, since it has rapidly become a key part of the pharmacological schemes used to treat Non-Hodgkin's lymphomas (NHLs). Moreover, due to its capacity to eliminate B lymphocytes, it has recently been applied in immune-mediated disorders [2].

Here, we will focus on the use of rituximab in the treatment of NHL, on the clinical issues associated with this therapeutic, and on the most recent advances in the field of lymphoma immunotherapy.

## 2. Tumor Antigens in NHL

When designing a therapeutic approach for NHL, cancer immunologists face the issue of selecting the best target antigen. Tumor antigens are traditionally divided in tumor-specific antigens (proteins that are uniquely expressed by cancer cells) and tumor-associated antigens (molecules that are expressed by cancer cells, although their expression is also found on normal cells) [3]. Ideally, an immune response against tumor antigens should destroy tumor cells without damaging normal cells. Thus, cancer-specific antigens would be the first choice. Unfortunately, true cancer-specific antigens, such as new proteins resulting from fusion oncogenes, are not frequent in NHL. Another important issue is to ensure that the chosen antigen does not mutate in a way that allows cancer cells to avoid destruction by the immune system [3].

The cell surface protein CD20 is a 33-kDa protein expressed by mature B cells and most malignant B cells, but not by pre-B cells or differentiated plasma cells [4–8]. In vitro studies have revealed that CD20 acts as a calcium ion channel [9, 10], and may also activate intracellular signaling through its ability to associate with the B-cell receptor (BCR) [11]. Interestingly, CD20's ability to induce cytosolic Ca^2+^ flux appears to be BCR dependent. Rituximab (Rituxan, Mabthera), is the first anti-CD20 monoclonal antibody approved by the Food and Drug Administration (FDA) (on November 26, 1994) for the treatment of relapsed or refractory, CD20+ follicular lymphoma (FL). It is a chimeric anti-CD20 antibody derived from the mouse mAb 2B8, targeting CD20 antigens, following replacement of the heavy and light chain constant regions with the corresponding regions of a human IgG1 mAb. Importantly, rituximab depletes both malignant and normal CD20^+^ B lymphocytes [4, 12, 13].

## 3. Rituximab's Mode of Action in Lymphoma Cells

Although the exact *in vivo* mechanisms of action for rituximab are not fully understood, the mechanisms of B-cell killing by this mAb have been exhaustively analyzed [14]. 

**Figure 1:**
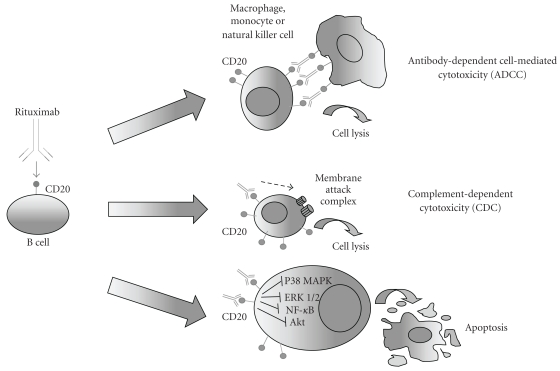
Schematic representation of the putative mechanisms mediating rituximab's anticancer activity in NHL cells. The anti-CD20 monoclonal antibody rituximab has several mechanisms of action, including antibody-dependent cellular cytotoxicity (ADCC), which involves recruitment of effector cells, mediated by Fc*γ* receptors; complement-dependent cytotoxicity (CDC); apoptosis induction.

Briefly, the major mechanism of rituximab-induced B-cell depletion involves antibody-dependent cell-mediated cytotoxicity (ADCC) and complement dependent cytotoxicity (CDC) [15]. Additionally rituximab was reported to directly induce apoptosis, inhibit B-cell proliferation, and to enhance the cytotoxic activity of chemotherapeutic agents [16] (Figure 1).

Rituximab-induced CDC is triggered upon rituximab binding to B cells with consequent initiation of the complement cascade starting from C_1_ activation. This mechanism causes osmotic lysis of neoplastic B cells [13, 14]. ADCC is triggered by the interaction between rituximab and the Fc receptor of natural killer (NK) cells [13, 14]. Once activated, NK cells release small proteins, including perforin and granzymes, which in turn form pores in the malignant B-cell membrane, and thus induce apoptosis or osmotic cell lysis. Finally, recent data demonstrate the novel role of rituximab as a signal-inducing antibody, and as a chemosensitizing agent, capable of negative regulation of major survival pathways [16].

Besides these mechanisms, rituximab's activity appears to be linked, at least in part, to its signaling via CD20. In this field, studies in B-NHL cell lines revealed several mechanisms involved in rituximab-mediated chemo/immunosensitization. Rituximab was shown to inhibit the p38 mitogen-activated protein kinase, nuclear factor-*κ*B (NF-*κ*B), extracellular signal-regulated kinase 1/2 (ERK 1/2), and Akt antiapoptotic survival pathways [17]. All of these effects result in upregulation of PTEN and of Raf kinase inhibitor protein (RKIP) [18], in the downregulation of antiapoptotic gene products, such as Bcl-2, Bcl-xL and Mcl-1, and, as a result, in chemo/immunosensitization [19]. In addition, treatment with rituximab inhibits the overexpressed transcription repressor Yin Yang 1 (YY1) [20]. YY1 downregulates Fas and DR5 expression and its inhibition leads to sensitization to Fas ligand and tumor necrosis factor-related apoptosis-inducing ligand- (TRAIL-) induced cell death [21].

Interestingly, recent studies also show that rituximab strongly affects BCR signaling [22]. Pretreatment of lymphoma cells or healthy B-cells with rituximab results in a time-dependent inhibition of the BCR-signaling cascade involving Lyn, Syk, PLC*γ*2, Akt, ERK, and calcium flux. Such inhibitory effects by rituximab are associated with a decrease in raft-associated cholesterol, inhibition of BCR relocalization to lipid rafts, and BCR downregulation. Since BCR signaling appears to be crucial for healthy and malignant B cell survival and expansion [23–25], this mode of action of rituximab could actually have an important role in mediating its anticancer activity.

The relative importance of each mechanism of action of rituximab is likely to vary with the type of tumor and the type of treatments that are administered together with this mAb. CDC and ADCC appear to be important to target leukemia/lymphoma cells circulating in the bloodstream [26]. Conversely, an immunological mechanism of action seems to be less important in the presence of nodal and extranodal involvement.

## 4. Rituximab's Applications in Hematological Malignancies

We will discuss here the current therapeutic applications of rituximab in indolent NHL, diffuse large B cell non-Hodgkin lymphoma (DLBCL), and in B-cell chronic lymphocytic leukemia (B-CLL). Although trials may have had endpoint definitions that are not always identical, almost all defined complete response (CR) as the complete disappearance of the symptoms and signs of lymphoma (including bone marrow clearing for >28 days), and partial response (PR) as a >50% decrease in the size or number of the lymphomas lesions, without any evidence of progressive disease for >28 days. CR and PR together represent the objective response (OR) rate [4, 27].

### 4.1. Follicular and Low Grade Lymphoma

Until the early 90's, the first-line therapy in symptomatic low-grade NHL was chlorambucil and prednisone [42]. Subsequently, several randomized trials showed the efficacy of rituximab in combination with other chemotherapeutic agents such as fludarabine (R-F), fludarabine, and cyclophosphamide (R-FC), fludarabine, cyclophosphamide and mitoxantrone (FCM-R), cyclophosphamide, vincristine, and prednisone (R-CVP), CVP plus mitoxantrone (R-CNOP), fludarabine, dexamethasone, and mitoxantrone (R-FND) as well as CHOP (R-CHOP) [28, 43–45] (Table 1). The clinical response rates of rituximab-containing regimens were encouraging, with an OR rates consistently around 95% and with a CR and PR rates ranging from 45% to 100%, and from 0% to 52%, respectively. 

Importantly, clinical data on the benefit of rituximab combined with chemotherapy has also become available in patients with relapsed or refractory indolent B-cell NHL. Also here, the results are very encouraging, with OR of 81% for R-CVP, 97% for R-FC, 88% for R-CHOP, and 95% for FCM-R respectively [4, 46].

Finally, the efficacy of rituximab monotherapy in patients with relapsed or refractory CD20-positive low-grade or follicular lymphoma was examined in noncomparative multicentre trials [33–35, 47–55]. The overall response rates were 38%–48% after a 4-week therapy with rituximab, and 57% after 8 weeks of rituximab administration. CR rates ranging between 3 and 17% were recorded in these studies.

Remarkably, studies show that, in FL, sequential administration of standard chemotherapy followed by rituximab induces molecular clearance (as detected by PCR for the Bcl-2/IgH rearrangement) in more than 70% of the patients [42, 43, 56]. The actual clinical impact of achieving a molecular response in FL still has to be determined, since long-term remissions have been reported also in patients with persistently detectable Bcl-2/IgH rearrangement [57]. Moreover this rearrangement may occasionally be found in healthy peripheral blood lymphocytes [58]. In fact, a recent study by van Oers and coworkers suggests that BCL-2/IgH polymerase chain reaction status at the end of induction treatment would not be predictive for progression-free survival in relapsed/resistant FL [59]. Nonetheless, the above-mentioned studies support the efficacy of rituximab in FL, and indicate its potential for treating minimal residual disease in this type of disorder. 

In summary, the current guidelines for the treatment of FL recommend that rituximab is administered in combination with standard chemotherapy in previously untreated stage III–IV FL, and at first relapse (at a dosage of 375 mg/m^2^ on day 1 of each chemotherapy cycle, for up to eight doses). Rituximab is recommended as a monotherapy for stage III–IV chemoresistant FL, or at second (or subsequent) relapse after chemotherapy (375 mg/m^2^ once weekly for four doses) (http://www.ema.europa.eu/docs/en_GB/document_library/Summary_of_opinion/human/000165/WC500097025.pdf).

### 4.2. DLBCL

After the disappointing results obtained with third-generation chemotherapy regimens in the United States, the CHOP regimen was reverted to as the standard of therapy. The efforts to introduce rituximab in the treatment of this aggressive hematological disease led to two essential clinical trials: the Mabthera International trial (MinT) [37] and the Groupe d'Etude des lymphomes de l'Adulte study (GELA) [36]. The first one involved young, the latter elderly, DLBCL patients. In the multicenter study conducted by Coiffier and colleagues, therapy using rituximab combined with standard CHOP chemotherapy demonstrated a higher efficacy than CHOP alone, in terms of both event-free survival at 2 years (57% versus 38%, *P* < .001), overall survival at 2 years (70% versus 57%, *P* < .01), and CR rate (76% versus 63%, *P* < .01). Likewise, the MinT study showed an increased OS of the combined rituximab-adding regimen, compared to standard therapy, from 84% to 93%. These results led to FDA approval of rituximab in combination with CHOP chemotherapy for previously untreated patients with DLBCL. Whether or not all patients need rituximab has been questioned. Studies from France and the American National Cancer Institute suggested that the benefit of rituximab would be observed in patients with tumors overexpressing Bcl-2. On the other hand, a recent report from the French group shows benefit in both Bcl-2-positive and Bcl-2-negative lymphomas using the method of competing risks [27, 60, 61]. Therefore, the question of whether molecular features should or will direct treatment decisions remains unanswered.

Finally, for recurrent DLBCL, the standard of care is salvage chemotherapy followed by high-dose chemotherapy with stem cell transplantation. Also in this setting, rituximab proved to be effective and has been incorporated into salvage chemotherapy regimens, since it may improve the overall response rate with ICE (ifosfamide, carboplatin, and etoposide) and DHAP (dexamethasone, high-dose cytarabine, and cisplatin) [62].

In summary rituximab is approved for previously untreated DLBCL patients in combination with CHOP chemotherapy and with salvage chemotherapy regimens in relapsed/refractory patients. The recommended rituximab dosage is 375 mg/m^2^ on day 1 of each chemotherapy cycle, for up to eight doses (http://www.ema.europa.eu/docs/en_GB/document_library/Summary_of_opinion/human/000165/WC500097025.pdf).

### 4.3. Rituximab and Autologous Stem Cells Transplantation for Advanced Stage DLBCL

Young high-risk patients with DLBCL achieving a complete remission after a complete course of chemotherapy are likely to benefit from autologous stem cell transplantation (ASCT) [27, 63]. Several studies are assessing the role of rituximab as part of high-dose regimens (HDT) pre-ASCT in DLBCL because of its effectiveness, limited toxicity, and its ability to deplete B cells. In this field, in a 2-year study by Khouri and colleagues evaluating the efficacy and safety of high-dose rituximab in combination with high-dose BEAM and ASCT, the OS was 80% for the study group compared to 53% for the control group [64]. Superior survival rates have also been reported for patients who become PCR negative for BCL2/JH rearrangements in peripheral blood or bone marrow compared with those who remain positive.

### 4.4. Rituximab Maintenance Therapy for FL and DLBCL

Despite the fact that rituximab used in combination with chemotherapy has been shown to prolong the survival of patients with NHL, residual lymphoma cells (which then become responsible for disease relapses) frequently remain [65]. As a matter of fact, NHL relapses continue to be an important clinical issue. Therefore, several randomized trials have been conducted in order to analyze the benefit of rituximab maintenance treatment in NHL [66–68]. The studies that were done for FL adopted different schemes for induction (rituximab 375 mg/m^2^ weekly × 4 in Ghielmini et al. and in Hainsworth et al.; CHOP or R-CHOP in van Oers et al.; fludarabine, cyclophosphamide, and mitoxantrone with or without rituximab in Forstpointner et al.) as well as for the maintenance treatment (375 mg/m^2^ intravenously weekly for 4 weeks at six-month intervals in Hainsworth et al.; 375 mg/m^2^ intravenously weekly for 4 weeks for Ghielmini et al.; 375 mg/m^2^ rituximab intravenously once every 3 months in van Oers et al.; 2 further courses of 4-times-weekly doses of rituximab after 3 and 9 months in Forstpointner et al.). However, overall, they unequivocally show that rituximab maintenance increases event-free survival (EFS) and duration of response in indolent NHL. In Ghielmini et al., at a median followup of 35 months, the median EFS was 12 months in the no-maintenance group versus 23 months in the prolonged treatment arm (*P* = .02) [69]. The authors reported that the difference was particularly notable in chemotherapy-naive patients (19 versus 36 months; *P* = .009) and in patients responding to induction treatment (16 versus 36 months; *P* = .004). In the study by van Oers et al., rituximab maintenance significantly improved EFS compared with observation (median, 3.7 years versus 1.3 years; *P* < .00), both after CHOP induction (*P* < .001) and R-CHOP (*P* = .003) [30]. The 5-year overall survival (OS) was 74% in the rituximab maintenance arm, and it was 64% in the observation arm (*P* = .07). Finally, also in the trial by Forstpointner and colleagues, response duration was significantly prolonged by rituximab maintenance, with the median not being reached in this evaluation versus an estimated median of 16 months in the observation group (*P* = .001) [31]. This beneficial effect was also observed when analyzing FL (*P* = .035) and mantle cell lymphoma (*P* = .049) separately.

Unlike in indolent NHL, rituximab maintenance therapy in DLBCL has failed to demonstrate benefit in the published clinical trials [38]. 

In conclusion, the current guidelines recommend the use of rituximab as a maintenance therapy only in relapsed or refractory follicular lymphoma responding to induction therapy with chemotherapy with or without rituximab. The recommended dosage of rituximab is 375 mg/m^2^ once every 3 months until disease progression or for a maximum of 2 years (http://www.ema.europa.eu/docs/en_GB/document_library/Summary_of_opinion/human/000165/WC500097025.pdf).

### 4.5. B-cell Chronic Lymphocytic Leukemia (B-CLL)

B-CLL is a heterogeneous disorder with a variable course (i.e., following diagnosis, survival ranges from months to decades) and risk factors such as age and performance status should be considered when selecting the most appropriate treatment option [70].

Rituximab monotherapy is generally not associated with sustained responses in B-CLL, possibly reflecting altered rituximab pharmacokinetics in patients with B-CLL [40, 70–73]. However, studies show that the addition of rituximab to fludarabine plus cyclophosphamide (FC) does improve clinical outcomes in B-CLL patients. The first study, known as CLL8, was conducted by the German CLL Study Group on 817 previously untreated B-CLL patients (ClinicalTrials.gov number, NCT00281918). The second trial, known as REACH, enrolled 552 patients with relapsed or refractory B-CLL following prior systemic therapy [40]. Both studies showed a benefit in terms of OS rates in the R-FC arm versus FC arm (86% versus 73 % in the CLL8 trial and 54% versus 45% in the REACH). In addition, the benefit of adding rituximab to chemotherapy in B-CLL was shown by several other trials [40, 74–77].

Interestingly, since rituximab plus FC represents the standard treatment for B-CLL, clinical studies compared the conventional regimen to rituximab plus low-dose FC (i.e., FCR-Lite) or to sequential FC and rituximab [49], since these alternative regimens are expected to be associated with less grade 3 or 4 neutropenia than the conventional R-FC regimen [5, 50].

The current international guidelines recommend that chemoimmunotherapy regimens with R-FC are preferred as the first-line treatment for advanced CLL (stage II–IV) in patients without del(17p) who are aged <70 years or aged >70 years without significant comorbidities. Among patients with relapsed or refractory disease, those with a long response (i.e., >3 years) can be retreated with one of the first-line treatment options. Various chemoimmunotherapy options are suggested for patients with a short response (i.e., <2 years) (e.g., rituximab may be administered in combination with FC or with CHOP). The recommended dosage of rituximab is 375 mg/m^2^ the day before starting chemotherapy, followed by 500 mg/m2 on day 1 of cycles 2–6 (National Comprehensive Cancer Network. NCCN clinical practice guidelines in oncology: non-Hodgkin's lymphoma).

## 5. Tolerability

Adverse events were reported in 84% of patients, receiving rituximab, during therapy or within the first 30 days following treatment [4, 35]. However, more than 95% of these events were described as mild to moderate in severity, of brief duration, and observed during the first infusion. The most common adverse effects were infusion-related reactions and lymphopenia. Ten percent of the patients reported severe fever, chills, infections, or other adverse effects. Serious adverse effects included severe infusion-related reactions, tumor lysis syndrome, mucocutaneous reactions, hypersensitivity reactions, cardiac arrhythmias, angina, and renal failure [4, 51].

These adverse events were less common during the subsequent rituximab administrations. One possible hematological adverse event is the reduction in peripheral B-lymphocyte counts, which can last for up to 6 months with a recovery period of 9 to 12 months [4, 35]. Nevertheless, the risk of serious opportunistic infections appears to be much lower than that reported with conventional therapy [4]. Interestingly, Bedognetti and coworkers have recently evaluated the impact of rituximab on the effectiveness of an antiflu vaccine in patients who had previously been treated with this mAb [52]. Due to the fact that disease status might affect immune response, only NHL patients without evidence of disease, who had completed rituximab no less than 6 months before the accrual, were selected for this evaluation. The study showed that patients who had previously received rituximab had a significantly lower seroconversion rate in response to the vaccine. Remarkably, while peripheral CD27- naïve B cells were present, Bedognetti et al. found a profound depletion in CD27+B memory cells, which may well explain the defective induction of antiflu immunity. Thus, concerns remain that patients who have been treated with the anti-CD20 mAb may be at risk for infections and that they may need careful monitoring.

## 6. Improving Rituximab Efficacy and Overcoming Resistance

Despite the expression of CD20 on their lymphoma cells, some patients exhibit primary resistance and do not respond well to this targeted antibody therapy. Moreover, an initially responsive lymphoma can subsequently become resistant to rituximab (secondary/acquired resistance). Several mechanisms have been reported that have the potential to contribute to reductions in rituximab efficacy. The identification of such mechanisms has allowed for the proposal of strategies to overcome these issues, and thus achieve better *in vivo* activity. Some of these mechanisms have been reviewed elsewhere [53]. Here, we will summarize some of the most recent and promising observations, and the related suggestions for therapeutic interventions.

- *Interfering with CD20 Downregulation/Shaving*. Initial* in vitro* observations suggested that CD20 would not be downregulated in the presence of anti-CD20 antibodies. Namely, the anti-CD20/CD20 complex was found to remain at the cell surface long enough to ensure cell killing by specific mechanisms. However, these observations may not be reproduced in *in vivo *settings. A recent report by Beers et al. showed that rituximab is able to induce CD20 internalization in a B-CLL mouse model [54]. Interestingly, these authors demonstrated that the degree of CD20 downmodulation correlates inversely with some types of NHL's susceptibility to rituximab. Namely, CLL and mantle cell lymphoma showed greater downmodulation of CD20 in response to rituximab than FL and DLBCL did, and were less responsive to treatment. Previous reports by Beum et al. described a “shaving reaction” in which mAb-CD20 complexes were “shaved” off CLL cells, by phagocytes, as the malignant cells circulated [78]. Whether the observed reduction in CD20 levels actually reflects shaving, or rather antigen masking by rituximab, remains unclear [79, 80]. Downregulation of CD20 access, irrespective of the underling cause, appears to be an important mechanism affecting rituximab efficacy, as antigen loss by malignant cells will prevent rituximab activity. New anti-CD20 mAbs (tositumomab-like) may be able to induce considerably less CD20 down-modulation than rituximab, and thus possibly be more effective (see below) [54]. It is also of interest that CD20 expression on lymphoma cells can be increased with HDAC inhibitors, such as valproic acid and romidepsin [81]. These were shown to transactivate the CD20 gene through promoter hyperacetylation and Sp1 recruitment. In line with these premises, HDAC inhibitors potentiated the activity of rituximab both *in vitro* and *in vivo* in murine lymphoma models.
- *Targeting CD20 Transcript Variants Associated with Resistance*. Henry and coworkers have recently identified an alternative CD20 transcript variant (ΔCD20) associated with resistance to rituximab [82]. This novel, alternatively spliced CD20 variant encodes for a truncated 130 amino acid protein lacking large parts of the four transmembrane domains, suggesting that ΔCD20 is a nonanchored membrane protein. ΔCD20 expression was detected in B-cell leukemias, B-cell lymphomas, and activated B cells, but not in healthy resting B cells. Finally, the authors went on to show that ΔCD20 is associated with resistance to rituximab, although the mechanism whereby this CD20 splice variant impairs the benefit of rituximab remains to be determined. The authors suggest that, given its selective expression in malignant (and activated) B-cells, ΔCD20 could become a therapeutic target, for instance for the development of antilymphoma vaccines. Whether this approach will prove effective remains to be assessed.
- *Preventing NK Cell-Mediated ADCC Exhaustion*. NK cell-mediated ADCC can be exhausted. Studies showed that NK cells can engage and kill 3-4 target cells in 16 hours. Thereafter, cells become exhausted, possibly due to a reduction in the available levels of perforin and granzyme B [83]. Indeed, incubation of NK cells with rituximab-coated target cells leads to CD16 (Fc*γ*RIIIa) downregulation and to upregulation of CD107a, a marker for degranulation and exhaustion [84, 85]. Finally, NK cell-mediated target cell killing was shown to become less efficient in the presence of high burdens of rituximab-opsonized lymphoma cells [86]. Remarkably, IL-2 treatment can restore NK cell-mediated ADCC. In line with this concept, Berdeja and coworkers found that systemic interleukin-2 and adoptive transfer of lymphokine-activated killer cells improves antibody-dependent cellular cytotoxicity in patients with relapsed B-cell lymphoma treated with rituximab [86]. In this context, recent studies showed that also complement components, such as C3b, can inhibit NK-cell mediated killing of mAb-opsonized lymphoma cells [55]. Importantly, C3 depletion by cobra venum factor, or the related drug (HC3-1496), appears to effectively overcome this mechanism and improve the activity of rituximab in lymphoma-bearing mice. Thus, overall, strategies aimed to improve NK cell activity could help enhance the efficacy of rituximab and should therefore be further investigated.
- *Enhancing CDC*. Studies showed that also complement can be depleted upon rituximab infusion in B-CLL patients [79]. Kennedy et al. found that fresh frozen plasma would then restore rituximab efficacy. More studies on this approach should be performed in order to confirm its viability. Another approach to enhance rituximab-induced CDC has recently been proposed by Wang and colleagues [87]. These authors observed that many tumors, including lymphomas, upregulate the expression of CD46, an inhibitory complement receptor. As a means to overcome this issue, they identify a recombinant adenovirus type 35 fiber knop protein (Ad35K^++^) which, when incubated with lymphoma cells, leads to CD46 downregulation and cooperates with rituximab in inducing CDC. In xenograft models with human lymphoma cells, preinjection of Ad35K^++^ dramatically increased the efficacy of rituximab, suggesting that the Ad35K^++^-based approach has potential implications in mAb therapy of NHL. Finally, Sato and colleagues have recently reported the identification of a novel CDC-enhancing variant of rituximab (113F) [88]. Compared to rituximab, 113F appeared to mediate highly enhanced CDC against primary CD20-expressing lymphoma cells in vitro. Moreover, these authors were able to establishe a human tumor-bearing NOD/Shi-scid-IL-2R*γ*(null) mouse model, in which human complement functions as the CDC mediator. Using this model, the authors demonstrated that 113F exerted significantly more potent antitumor effects than rituximab.
- *Improving Phagocytosis Through CD47 Blockade*. Chao and colleagues have recently shown that multiple B-cell NHL subtypes, including DLBCL, FL, and B-CLL, exhibit increased levels of CD47, a transmembrane protein which activates SIRP1a in phagocytic cells [89]. This results in initiation of a signal transduction cascade which leads to phagocytosis inhibition. These authors demonstrate that CD47 overexpression correlates with worse prognosis. Blocking anti-CD47 antibodies promote phagocytosis of NHL cells and cooperate with rituximab both *in vitro* and *in vivo* in murine NHL xenotransplant models. Again, whether this approach will prove useful in humans remains to be assessed.
- *Topical* IFN-*αDelivery*. Finally, Xuan and colleagues have proposed an approach to target IFN-*α* molecules to lymphoma sites by constructing a fusion protein consisting of IFN-*α* and an anti-CD20 mAb [90]. IFN-*α* has potent immunostimulatory properties and antiproliferative effects in some B-cell NHLs, but its systemic administration is frequently associated to significant toxicity. The CD20-IFN-*α* fusion proteins showed efficient anticancer activity against an aggressive rituximab- resistant human CD20+ murine lymphoma (38C13-huCD20) and a human B-cell lymphoma (Daudi). Further experimentation with this administration method is warranted to assess its applicability in patients.
- *Rituximab Mutants with Proapoptotic Activity*. In order to improve rituximab anticancer activity, Li and colleagues modulated the binding property of this mAb by introducing several point mutations in its complementarity-determining regions [91]. These authors found that the CDC potency of such CD20 mAbs was independent of the off-rate. However, they were able to identify a rituximab triple mutant (H57DE/H102YK/L93NR) with an extremely potent apoptosis-inducing activity. This triple mutant efficiently initiated both caspase-dependent and-independent apoptosis, and exhibited potent *in vivo* activity even in a rituximab-resistant lymphoma model. These modified versions of rituximab hold promise as new therapeutic agents for B-cell lymphomas, although their efficacy in patients still has to be assessed.
- *Combining Rituximab with Other mAbs*. Rituximab activity in NHL as a single agent is limited, especially when administered to pretreated patients. However, combining rituximab with chemotherapy does achieve significantly better outcomes than chemotherapy alone. In addition, strategies to use two mAbs have also been proposed. Combinations such as anti-CD20 plus anti-CD22, anti-CD20 plus anti-HLA-DR, anti-CD20 plus anti-TRAIL-R1, anti-CD20 plus anti-CD80 have been evaluated preclinically and/or clinically, showing enhanced antitumor activity both *in vitro* and *in vivo* [92–94]. An interesting approach to achieve the benefit of multiple targeting in NHL consists of the generation of multivalent antibodies using the so-named Dock-and-Lock (DNL) method, which enables site-specific self-assembly of two modular components with each other, resulting in a covalent structure with retained bioactivity [95]. Using this approach, Rossi and colleagues generated bispecific anti-CD20/CD22 hexavalent antibodies with promising antilymphoma activity *in vitro* and *in vivo* [96, 97]. Interestingly, in a recent study, these authors were able to correlate the strong direct cytotoxicity of the anti-CD20/CD22 hexavalent antibodies, compared to their bivalent parental antibodies, with their increased ability to upregulate PTEN, phospho-p38, and cyclin-dependent kinase inhibitors, such as p21, p27 and Kip2 [98].

## 7. Other mAbs for NHL

In addition to the above-mentioned strategies aiming to improve rituximab activity, numerous research efforts have led to new mAbs directed against different target antigens and to the development of radioimmunoconjugates. The most promising newer therapeutics are listed below.

- Epratuzumab: a humanized IgG1 anti-CD22 antibody. It induces ADCC and CDC in preclinical studies. Phase I/II studies demonstrated objective responses in relapsed/refractory FL (24%) [99], and in DLBCL (15%) [100], without dose-limiting toxic effects.
- Galiximab: a primatised anti-CD80 (IgG1*λ*) mAb with human constant regions and primate (cynomologus macaque) variable regions [101]. CD80 is a costimulatory molecule involved in regulating T-cell activation. It is transiently expressed on the surface of activated B cells, dendritic cells, and T cells of healthy individuals [102]. Additionally, a variety of lymphoid malignancies constitutively express CD80, making this antigen a suitable target [103]. A phase-I/II study showed that GALIXIMAB is able to enhance rituximab antitumor activity in previously untreated NLH patients, with a response reported in 70% of patients [104].
- Alemtuzumab (Campath): a humanized monoclonal antibody against CD52 (an antigen expressed by normal and malignant B- and T-lymphocytes, monocytes, and NK cells). It is indicated for the treatment of patients with B-CLL refractory to fludarabine (ORR of 56%) [105], for advanced-stage mycosis fungoides/Sezary syndrome [106], and for relapsed or refractory peripheral T-cell lymphomas [107, 108]. Notably, although clinically effective, this mAb induces a dramatic decrease in CD4+ and CD8+ T lymphocytes and thus strongly increases the risk of infections.
- Apolizumab (Hu1D10): a humanized anti-HLA-DR antibody that induces CDC, ADCC, and apoptosis. HLA class II antigens are expressed at the surface of professional antigen presenting cells, including B cells. They are involved in antigen presentation and in promoting cell proliferation. Thus, mAbs against HLA-DR inhibit B-cell proliferation and induce apoptosis through activation of the extrinsic apoptotic pathway. Recently, this type of approach has shown promising results in B-cell malignances [109]. Single agent therapy APOLIZUMAB in previously untreated B-CLL patients showed an ORR of 83% [110]. Moreover, the combination of APOLIZUMAB and rituximab in relapsed/refractory B-cell lymphoma and B-CLL showed an ORR of 42% [111].
- *Radioimmunotherapy:* this type of treatment involves the administration of an antibody linked to a radioisotope. This approach permits the targeting of the radioactive isotopes to cancer tissues and is especially interesting as it allows for killing neighboring cancer cells that either are inaccessible to the antibody or express insufficient antigen for the antibody to bind in adequate quantities. Two anti-CD20 radioimmunoconjugates are approved for use in patients with relapsed or refractory follicular or low-grade lymphoma:
  1. Yttrium-90: labelled ibritumomab tiuxetan (zevalin),
  2. iodine-131: labelled tositumomab (bexxar).

These therapeutics hold great promise for the treatment of NHL and their usefulness has recently been confirmed by several clinical trials [112–122]. 

About 80% of patients with follicular or low-grade lymphomas respond to treatment with Zevalin, with 20 to 30% achieving a CR. Interestingly, the duration of response appears to exceed 3 years in about 25% of patients [123]. The benefit of adding a radioisotope to the antibody was confirmed in a study enrolling patients with indolent NHL that were refractory to rituximab. In this study, Zevalin showed a 74% response rate and 15% of CR [115]. Additionally, as compared to rituximab, Zevalin produces higher response rates among patients with follicular or low-grade lymphoma who have not previously received antibody-based treatments (ORR 80% versus 56%, *P* = .002; CR 30% versus 16%, *P* = .04 [115]). Finally Zevalin also appears to be effective against some diffuse large B-cell lymphomas, and mantle-cell lymphomas, when used in sequence with chemotherapy (ORR of 53% versus 19%; OS 22.4, versus 4.6, resp.) [117].

Similar results are obtained with Bexxar. In particular in patients with NHL refractory to standard chemotherapy, treatment with Bexxar resulted in CR in 20% of patients [124]. Additionally, in one study, 95% of patients with NHL had responses to 131I-labeled tositumomab used as initial treatment, with 75% demonstrating CR [125]. Finally, the results of a recently completed study (ClinicalTrials.gov number, NCT00006721), comparing CHOP followed by 131I-labeled tositumomab to rituximab plus CHOP for the initial treatment of FL, is predicted to redefine standard therapy for this disorder.

Importantly, there are other radiolabeled immunotherapeutics for NHL that are currently under evaluation [112, 114, 126–130]. These include

- LL_2_ anti-CD22, conjugated to either _131_I or _90_Y; Lym-I;
- anti-HLA-DR, conjugated to _90_Y or _67 _Cu;
- rituximab, conjugated to _211_At, _186_Re, or _227_Th;
- anti-CD19 mAb conjugated to _90_Y.

## 8. Conclusions and Perspectives

Combining rituximab with chemotherapy has proven to be an effective treatment for both indolent and aggressive forms of NHL. The same type of treatment can be used in patients with B-CLL, although its efficacy in this disorder appears to be lower. In addition, it has also been demonstrated that using rituximab alone as a maintenance therapy improves the prognosis and extends disease-free survival in FL. Although a standard scheme for rituximab maintenance therapy has not been established yet, it is currently under investigation and the ongoing studies will establish the most effective regimen.

For patients in which treatment with rituximab has not given the expected results, autologous stem cell transplantations have shown promise. It has been demonstrated that using a cycle of rituximab in association with stem cell transplantations and after it as maintenance therapy, yields better results than transplant alone.

Radiolabeled antibodies may be effective in rituximab-resistant and chemotherapy-resistant disease, but their clinical use is still limited when compared to that of unlabeled mAbs. Recent data suggest that sequential radioimmunotherapy after chemotherapy may have significant clinical value. Additionally, novel monoclonal antibodies are under development. If these will prove to be more effective than rituximab will have to be assessed by randomized comparative trials.

Overall, the results obtained with antibody-based therapeutics in NHL are clearly highly promising. They herald the advent of therapeutic strategies based on targeted agents that will likely be more effective and, at the same time, less toxic than traditional chemotherapy-based treatments.

##  Grant Support

Alessio Nencioni is supported by the Associazione Italiana per la Ricerca sul Cancro (AIRC) and by the University of Genoa.

## Figures and Tables

**Table 1:** Principal clinical trials of chemotherapy plus Rituximab versus chemotherapy alone in NHL.

Lymphoma Subtype	Treatment	Patients (no.)	% Overall response rate (*P* value)*	Median Follow-up (mo.)	Reference
Follicular	CVP versus R-CVP	321	57 versus 81 (<.001)	53	Marcus et al. [28]
Follicular	CHOP versus R-CHOP	428	90 versus 96 (=.011)	18	Hiddemann et al. [29]
Follicular	CHOP versus R-CHOP	465	72.3 versus 85.1 (<.001)	39,4	van Oers et al. [30]
Follicular	FCM versus R-FCM	176	71 versus 95 (=.01)	26	Forstpointner et al. [31]
Follicular	MCP versus R-MCP	201	75 versus 92 (=.009)	47	Herold et al. [32]
relapsed/refractary low grade	R	37	46	13,4	Maloney et al. [33]
relapsed/refractary low grade	R	30	47	19	Feuring-Buske et al. [34]
relapsed/refractary low grade	R	166	48	19,5	McLaughlin et al. [35]
DLBCL	CHOP versus R-CHOP	399	63 versus 76 (=.005)*	24	Coiffier et al. [36]
DLBCL	CHOP versus R-CHOP	824	84 versus 93 (=.0001)**	34	Pfreundschuh et al. [37]
DLBCL	CHOP versus R-CHOP	632	57 versus 67 (=.05)**	42	Habermann et al. [38]
DLBCL	CHOP versus R-CHOP	122	75 versus 94 (=.0054)	18	Lenz et al. [39]
B-CLL	FC versus R-FC	552	58 versus 69.9 (=.0034)	25	Robak et al. [40]
B-CLL	FC versus R-FC	817	82.5 versus 87.2 (=.012)**	37,7	CLL8- German CLL Study Group*** [41]

*CR, CR-unconfirmed, partial response.

**CR rate.

***3-year OS.
